# The Role of Neurotrophins in Multiple Sclerosis—Pathological and Clinical Implications

**DOI:** 10.3390/ijms131013713

**Published:** 2012-10-22

**Authors:** Alicja Kalinowska-Lyszczarz, Jacek Losy

**Affiliations:** 1 Department of Neurochemistry and Neuropathology, Chair of Neurology, Poznan University of Medical Sciences, 49, Przybyszewskiego st., 60-355 Poznan, Poland; 2 Heliodor Swiecicki University Hospital, 60-355 Poznan, Poland; 3 Department of Clinical Neuroimmunology, Chair of Neurology, Poznan University of Medical Sciences, Poland; E-Mail: jlosy@ump.edu.pl; 4 Neuroimmunological Unit, Institute of Experimental and Clinical Medicine, Polish Academy of Sciences, 60-355 Poznan, Poland

**Keywords:** multiple sclerosis, neurotrophins, neuroimmunology, peripheral blood mononuclear cells, neuroprotective autoimmunity

## Abstract

Multiple sclerosis (MS) is a chronic inflammatory demyelinating and neurodegenerative disease of the central nervous system (CNS) with unknown etiology. It was recently suggested that autoimmunity, which had long been considered to be destructive in MS, might also play a protective role in the CNS of MS patients. Neurotrophins are polypeptides belonging to the neurotrophic factor family. While neurotrophins mediate cell survival and proliferation in the nervous system, they are also expressed within peripheral blood mononuclear cells fraction (PBMCs) of immunological system. In MS additional neurotrophic support from PBMCs might compensate relative neurotrophins deficiency in the damaged CNS tissue that needs to be repaired. Failure to produce the adequate neurotrophins concentrations might result in decreased protection of the CNS, consequently leading to increased atrophy, which is the main determinant of MS patients’ end-point disability. There are several lines of evidence, both from clinical research and animal models, suggesting that neurotrophins play a pivotal role in neuroprotective and neuroregenerative processes that are often defective in the course of MS. It seems that neuroprotective strategies might be used as potentially valuable add-on therapies, alongside traditional immunomodulatory treatment in multiple sclerosis.

## 1. Introduction

Multiple sclerosis (MS) is a chronic inflammatory disease of the central nervous system (CNS), with yet unknown etiology, leading to formation of disseminated demyelinating lesions and accompanied by axonal degeneration. The disease affects predominantly young women, with the usual 2:1 ratio, as presented in a number of representative studies [1–3]. In its most typical form, namely relapsing-remitting MS (RRMS), it presents as attacks of diverse neurological symptoms, usually—but not necessarily—followed by conversion to the progressive phase and accumulation of disability in patients [4]. Disease severity varies from one patient to another, including benign, monosymptomatic cases, but also malignant and rapidly progressive ones. Median time from disease onset to progression depends mainly on the age of onset: the older the age, the shorter the time to secondary progression [1]. Several therapies are currently approved for MS treatment, and a considerate number is still under clinical trials. Their primary effect is the reduction of the annual relapse rate and of the radiological activity of the disease, as described by the number of new gadolinium-enhancing lesions and new T2-hyperintense lesions demonstrated in subsequent magnetic resonance imaging (MRI) scans; yet, their effect on disease progression is not satisfactory. Ever since the first descriptions of MS, the majority of research in the field has been focusing on its inflammatory component. In the recent years attention has also been drawn to its neurodegenerative aspect. In this context studies on the potentially protective role of neurotrophic factors have emerged in MS research. In this review the role of neurotrophins in multiple sclerosis is presented and potential future therapeutic options are discussed.

## 2. Biology of Neurotrophins

**Figure 1:**
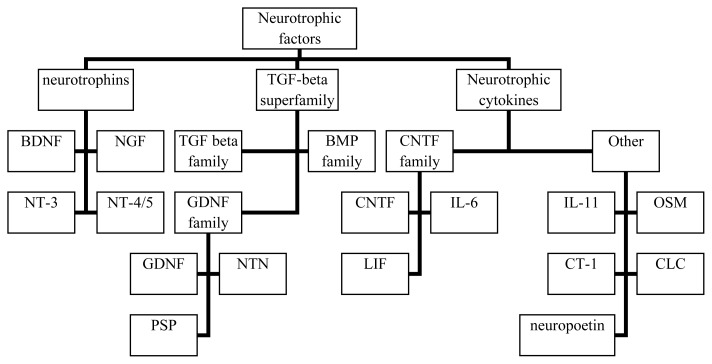
Neurotrophic factor family classification. NGF: nerve growth factor, BDNF: brain-derived neurotrophic factor, NT-3: neurotrophin 3, NT-4/5: neurotrophin 4/5, TGF beta: transforming growth factor beta, GDNF: glial derived neurotrophic factor, BMP: bone morphogenic proteins, NTN: neurturin, PSP: persephin, CNTF: ciliary neurotrophic factor, IL-6: interleukin 6, LIF: leukemia inhibitory factor, IL-11: interleukin 11, OSM: oncostatin M, CT-1: kardiotrophin-1, CLC: cardiothrophin*-*like cytokine.

Neurotrophins are secretory polypeptides belonging to the neurotrophic factors’ family (see Figure 1). During the development of the nervous system, neurotrophin deprivation serves as a physiological mechanism of neuronal elimination [5]. In the adult CNS, neurotrophins play a protective role towards specific neuronal populations. They also facilitate synaptic transmission and plasticity [6–9] that are crucial for memory and regeneration processes.

**Figure 2:**
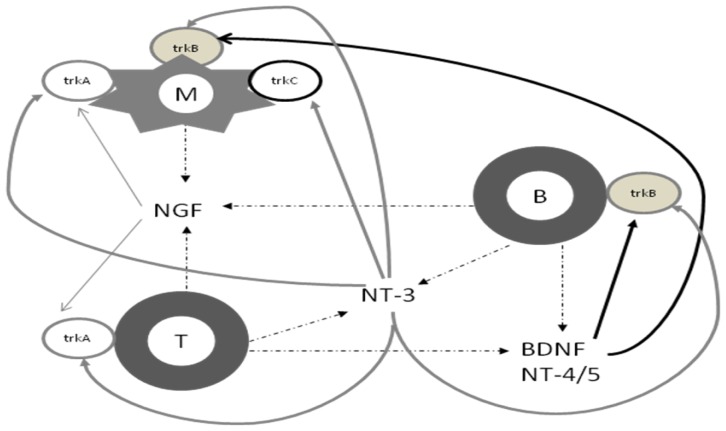
Interactions between neurotrophins and the immune cells. NGF: nerve growth factor, BDNF: brain-derived neurotrophic factor, NT-3: neurotrophin 3, NT-4/5: neurotrophin 4/5, trk: tropomyosin related kinase receptor, M: macrophage, T: T lymphocyte, B: B lymphocyte. Dotted lines indicate secretion. Straight lines indicate affinity towards receptors.

Nerve growth factor (NGF) was the first neurotrophin to be identified [10]. The family also includes brain-derived neurotrophic factor (BDNF), neurotrophin-3 (NT-3) and neurotrophin 4/5 (NT-4/5), otherwise called neurotrophin-4 (NT-4) or neurotrophin-5 (NT-5), with so far not identified in mammals neurotrophin-6 (NT-6) and neurotrophin-7 (NT-7) [11]. All neurotrophins are synthesized as precursor proteins and become biologically active by metalloproteinase cleavage [12]. Neurotrophins bind with high affinity to tropomyosin related kinase (trk) receptors [13], and—with low affinity—to p75^NTR^ receptors that belong to tumor necrosis factor (TNF) receptor superfamily [14]. Different neurotrophins bind preferentially to respective trk receptors [15], see Figure 2, which results in trk dimerization, trans-autophosphorylation and subsequent activation of such intracellular signaling pathways as Ras/ERK and Akt/PI3K, both promoting cell survival [11,13]. On the other hand, the p75^NTR^ molecule can act as a co-receptor for trk receptors, or it can induce an independent signaling pathway, promoting either survival (via NF-κB), or apoptosis (Rac1/Jun N-terminal kinase, JNK) [16,17]. The p75^NTR^ receptor is otherwise known as TNF receptor type 2 (TNFR-2). It is a known fact that disruption of TNF signaling pathway occurs in a number of autoimmune diseases, including MS. Interestingly, TNFR-2 pathway is an attractive drug target for autoimmune diseases, since its expression is restricted only to certain immune-cell populations, which could mean less side-effects than with the use of TNF-alpha alone, that acts on both, ubiquitously expressed TNFR-1 and more limited TNFR-2 [18]. The link between neurotrophins and TNF pathway obviously requires special attention. The biological results exerted by neurotrophins depend on their concentrations, receptor affinity and on their structure (pro-neurotrophins have higher affinity towards p75^NTR^ and promote apoptosis) [12,19]. Neurotrophins can also co-operate with other receptors or ion channels. Interactions between trk receptors and voltage-gated potassium channels or NMDA receptors have been described [20,21]. The p75^NTR^ can act as a co-receptor for the Nogo receptor that is known to inhibit axonal growth by interactions with MAG protein [22,23].

## 3. Neurotrophins and the Immune System

The nervous and the immune systems are interconnected at several levels. Neurotrophins may serve as a fine example of this complicated relation. Under physiological conditions the main source of neurotrophins within the CNS is the neuronal population, and so is their main target. However, neurotrophins are also expressed within the cells of the immunological system, namely peripheral blood mononuclear cells (PBMCs), as presented in Figure 2 [24–26]. Immune cells also express neurotrophin receptors: p75NTR and trkA are expressed on T cells and macrophages, trkB—on B-cells and macrophages, and trkC on macrophages only [24]. The ability of immune cells to secrete neurotrophins at the injury site within the CNS could be used as a potential therapeutic regimen. Under pathological conditions, as in MS, the additional neurotrophic support from PBMCs could compensate the relative neurotrophin deficiency in the damaged brain [27,28]. In MS PBMC-derived neurotrophins could play a neuroprotective role directly, binding to trk receptors on neurons within the CNS and promoting their survival, or indirectly by interacting with the neurotrophin receptors on the immune cells, regulating their functions (proliferation, immunoglobulin production) and determining their survival or apoptosis [15,29,30].

## 4. Neuroinflammation, Neurodegeneration and Neuroprotective Autoimmunity in MS

Traditionally, MS is considered to be caused by the autoreactive T-cells that become activated in the periphery, cross the blood-brain barrier (BBB) into the CNS and become re-activated by antigen presenting cells (APCs), which leads to a cascade of destructive inflammatory reactions [31]. Consequently, most of the therapies approved or tested for MS are based on their anti-inflammatory properties, and these are only partially effective. Neurodegeneration accompanies the inflammatory process in MS and it is still debated whether it is secondary to myelin sheath destruction, or if it progresses independently [32]. The most radical concept is that MS is in fact primarily neurodegenerative, whereas autoimmune reaction is only a response to the degenerative process [33,34]. This concept was supported by the elegant neuropathological study by Barnett and Prineas in 2004, where they showed that the earliest structural change in the newly forming demyelinating lesions was oligodendrocyte apoptosis, that preceded lymphocyte infiltration and phagocyte activation [33]. Apoptotic oligodendrocytes within new MS plaques were also observed by Lucchinetti *et al.* [35]. The cause of oligodendrocyte destruction has so far not been established, however, its early appearance suggests that at least in some MS cases autoimmune reaction may be triggered as a secondary phenomenon. While the primary neurodegenerative origin of MS may be considered controversial, autoimmunity does involve a protective component. The examples include CD4+ Th2 cells-derived anti-inflammatory cytokines, macrophage-derived epidermal growth factor (EGF), which has myelinoand oligodendrocyte-trophic properties, and secretion of neurotrophins by peripheral blood mononuclear cells (PBMCs). Also, clinical disease activity does not correlate with the total inflammatory lesion load measured with the use of MRI. Moreover, endpoint disability in MS patients is associated with brain atrophy measures, and not with demyelinating lesions’ volume [36,37].

Another interesting issue is the dual role that astrocytes may play in MS pathogenesis. Aside their structural and regulatory role within the CNS, they can be activated to exhibit immune cell functions. They are capable of acting as antigen presenting cells, providing environment for T cell activation, inhibiting myelin repair and enhancing immune reaction [38]. On the other hand, they can support oligodendrocyte and axonal regeneration, among many by inducing neurotrophin (BDNF, NT-4) synthesis [39].

Given the striking heterogeneity of MS and the limited efficacy of immunosuppressive/immunomodulatory agents, one should consider that it might be the defective neuroprotective and neuroregenerative properties that determine the disease course. So far, this aspect of MS has not been fully explored. However, more and more studies on this subject emerge.

## 5. Neurotrophins and MS Pathology

In 2002 Standelmann *et al.* showed that in MS-diseased brains BDNF was expressed not only in neurons, but also in T lymphocytes and macrophages, especially in the perivascular spaces, while this was not observed in healthy controls [40]. Immune cell BDNF expression was positively correlated with the inflammatory activity of demyelinating lesions. In the same study BDNF receptor, trkB, was found on neurons in close proximity of MS plaques and on reactive astrocytes within lesions. Interestingly, the highest concentrations of neurotrophic factors and their receptors in the CNS of MS patients are found within the active edge of the demyelinating plaques, where axons are at risk of bystander destruction adjacent to the neuroinflammatory core. These axons, which could be perceived as the ischaemic stroke “penumbra” equivalent, can still be salvaged with the adequate neuroprotective processes being activated [27,28]. If neuroprotective strategies fail, the area of damage becomes vaster, hence the increase of the CNS atrophy, which remains the main determinant of MS patients long-term disability. In this context it is highly probable that immune cells releasing neurotrophins into the periplaque area may in fact play a neuroprotective role. The fact that neurotrophin reactivity is higher in MS plaques can be interpreted as the protective aspect of the inflammatory process. One could expect that the additional supply of neurotrophins, by immune cells or pharmacologically, will exert a neuroprotective effect, reflected by a better clinical outcome in patients.

The first observation that raised hopes for neurotrophic factors as potential therapeutic agents in MS was finding that leukaemia inhibitory factor (LIF), belonging to the neurotrophic factor family, reduced clinical severity and promoted oligodendrocyte survival in mice suffering from an animal equivalent of MS, namely experimental autoimmune encephalomyelitis [41]. LIF was successful no matter if administered systemically at immunization, before disease onset, or after the disease has already developed. This observation confirmed that neurotrophic factors act by facilitating regeneration, and not by suppressing the inflammatory reaction which is not present before the disease becomes clinically active.

There are a number of studies on neurotrophins in animal models, which show that administration of neurotrophins enhances survival of injured neurons in EAE and other neuronal injury models [42,43]. The evidence in human disease is less extensive. It is worth mentioning that one can assess the neurotrophic potential by several means and different approaches have been used by different researchers. Neurotrophin concentrations can be measured directly in PBMCs lysates with the ELISA method. One can also estimate neurotrophin mRNA expression in PBMCs, or measure the secretion of activated immune cells in cultures. In a number of studies neurotrophin levels were estimated in CSF samples. However, in our opinion this seems less useful, since CSF neurotrophin concentrations are determined mainly by the neuronal pool. Consequently, the CSF neurotrophins concentrations may in fact reflect atrophy progression, and not explain it. Importantly, there are no normative values for neurotrophins concentrations in the body fluids.

From a clinical standpoint, the role of neurotrophins in MS is undoubtedly complex. So far the most abundant literature on neurotrophins’ function in MS is available for BDNF. However, the studies that were published often present obviously conflicting results. During remission BDNF levels were shown to be unchanged [44], decreased [45–47] or increased [48–50]. Data seems consistent on the fact that RRMS patients who experience relapse and are not treated with immunomodulation have higher BDNF levels [44,51]. On the contrary, these patients present lower BDNF levels at baseline, most likely because of the defective CD40-dependent stimulation, which is restored by IFN-beta [52].

So far it has been shown that PBMC-derived BDNF is related to axonoprotective potential in MS patients [45,53,54]. Our group observed that lower immune-cell NT-3 is associated with more advanced brain atrophy in RRMS patients [55]. We have also found that in RRMS patients cognitive deficit is related with immune-cell beta-NGF [56]. Similarily, Patanella *et al*. described correlations between lower PBMC-derived BDNF levels and worse performance in neuropsychological tests of MS patients [57]. A protective function of C rs2030324 BDNF allele was described in the context of visual information processing, thought to be related to thalamic volume [58].

## 6. Implications for Therapy

The findings that are mentioned above, although they should be interpreted cautiously, may have important implications for MS therapy. Neuroprotective properties towards neurons and oligodendrocytes, and immunomodulatory potential of neurotrophins raise hopes for their therapeutic application.

Since neurotrophins have a relatively short (less than two minutes) plasma half-life [59,60] it is not possible to deliver them systemically. Neurotrophins cannot cross the human blood-brain barrier unless they are modified, for instance using chimeric peptide brain drug targeting technology [61]. Other options that are worth exploring would be trk receptors agonists, selective trk monoclonal antibody or small-molecule trk agonists and, finally, a cellular approach of the adoptive transfer of activated autoimmune cells, modified in vitro to secrete neurotrophins when reinjected [27]. *In vivo* gene therapy with viral vectors injected directly into neurons is yet another option, and such attempt already passed phase I trial for treatment of Alzheimer’s disease (AD) [62]. Indeed, several attempts have been made to use neurotrophin-related pathways in therapy for other CNS disorders with a degenerative component, in which decreased neurotrophic activity was described, namely AD, Parkinson’s disease (PD), amyotrophic lateral sclerosis (ALS), and Huntington’s disease [63–67]. Interestingly, some of the immunomodulating agents currently used for MS, namely interferon beta, glatiramer acetate (GA) and—still under clinical trials—alemtuzumab, have been shown to increase serum and/or immune-cell levels of BDNF in MS patients, which was suggested as one of the possible modes of actions for these therapies [46,47,68]. For many years GA effects were thought to be mediated substantially by induction of a shift of GA-reactive T cells from Th1 to Th2. However, it was shown that GA-reactive T cells release BDNF locally in the CNS and thus exerting a neuroprotective effect [69]. Glatiramer acetate was also shown to augment NT-3 and NT-4 expression by T cells and resident CNS cells (neurons, astrocytes) in EAE models [70]. On the other hand, interferon beta was shown to promote NGF secretion early in the course of MS [71]. Laquinimod, which is a novel oral immunomodulatory drug developed for MS therapy—still under clinical trials, has so far demonstrated some beneficial effects in RRMS patients. Aside from its immunomodulatory mode of action, it has been shown to prevent EAE-induced BDNF loss and restore it in the chronic stage of the disease in mice [72]. Also, in MS patients, laquinimod administration resulted in the increase of serum BDNF levels [73].

The precise mechanisms in which immunomodulatory agents work in MS in not known, and their efficacy is largely established based on clinical trials. It may be so that the neuroprotective component of their mode of action is at least partially responsible for their clinical efficacy.

## 7. Concluding Remarks

In the healthy adult CNS neurons are the main source of neurotrophins and the role of immune cell-derived neurotrophic factors is most probably marginal, especially since it is only the activated immunological cells that can cross the blood-brain barrier and they do not get activated in the undamaged brain. On the contrary, in MS we observe dynamic interactions between the nervous and immunological systems.

It seems crucial to identify the mechanism in which PBMCs are stimulated to produce neurotrophins and release them into the plaque area. Such knowledge would be a breakthrough finding in context of therapy for MS and other CNS pathologies where neuroprotective-neuroregenerative strategies are needed. Shibata *et al*. (2003) showed that stimulation of human macrophages with CD40L ligand increases their neurotrophic activity, resulting in enhancing protein synthesis, promoting neurite growth and facilitating synaptic activity of rat cortical neurons cultured in media conditioned with the stimulated human macrophages [74]. CD40L is expressed on activated T cells, on cerebral endothelium [75] and on astrocytes [76]. Therefore, after crossing the BBB macrophages could be activated by all these cells to produce neurotrophins. However, in many RRMS patients, regeneration processes become insufficient and the disease progresses to the secondary progressive stage. One possible explanation would be that the neuroprotective function of PBMCs is inhibited because of the proinflammatory shift of the immunological reaction. This phenomenon could account for the antigen non-specific immunological defect that leads to MS development under conditions in which subjects with no such defect would not develop the disease.

Identification of the exact role that neurotrophins play in MS pathology could serve not only for potential therapeutic approaches, but also in the diagnostic field. Flooded with a vast number of communications on detailed interactions in MS immunology, we are lacking information that could be useful in clinical practice. Currently there are no valid methods evaluating neuroprotection in patients. Standard MRI measures, with the exception of the T1-hypointense “black holes” volume, correlate poorly with axonal loss. CSF biomarkers are difficult to obtain, given the patients’ reluctance to repetitive lumbar puncture procedures. At the moment there are no validated biomarkers that could be used for MS diagnosis, monitoring and prognosis of disease progression. Neurotrophins could be a plausible candidate for MS-related biomarkers, especially in light of evidence suggesting their association with brain atrophy markers and cognitive dysfunction in MS patients (see earlier). For instance, they might serve as surrogate markers of patients’ neuroprotective potential that is correlated with brain atrophy.

Undoubtedly, there is more and more evidence for the role of neurotrophins as possible mediators of the neuroprotective function that inflammatory cells may exert in MS. More studies, especially observations in MS patients and not animal models, are needed in order to clarify this aspect and start considering neurotrophins as potential biomarkers and/or add-on therapies to traditional immunomodulation in multiple sclerosis patients.
